# URG4/URGCP enhances the angiogenic capacity of human hepatocellular carcinoma cells *in vitro* via activation of the NF-κB signaling pathway

**DOI:** 10.1186/s12885-015-1378-7

**Published:** 2015-05-07

**Authors:** Sizhong Xing, Bing Zhang, Ruixi Hua, William Chi-shing Tai, Zhirong Zeng, Binhui Xie, Chenghui Huang, Jisu Xue, Shiqiu Xiong, Jianyong Yang, Side Liu, Heping Li

**Affiliations:** 1 Guangdong Provincial Key Laboratory of Gastroenterology, Department of Gastroenterology, Nanfang Hospital, Southern Medical University, Guangzhou, 510000 P.R. China; 2 Department of Gastroenterology, the First Affiliated Hospital of Sun Yat-sen University, Guangzhou, 510000 P.R. China; 3 Department of Internal Medicine, Baoan People’s Hospital, Shenzhen, 518101 P.R. China; 4 Department of Medical Imaging, the First Affiliated Hospital of Sun Yat-sen University, Guangzhou, 510000 P.R. China; 5 Department of Oncology, the First Affiliated Hospital of Sun Yat-sen University, Guangzhou, 510000 P.R. China; 6 Center for Cancer and Inflammation Research, Institute of Integrated Bioinformedicine and Translational Science, Hong Kong Baptist University, Hong Kong, S.A.R. China; 7 Department of Hepatobiliary Surgery, the First Affiliated Hospital of Gannan Medical University, Ganzhou, 341000 P.R. China; 8 Department of Biochemistry, University of Leicester, Leicester, UK

**Keywords:** URG4/URGCP, Hepatocellular carcinoma, Angiogenesis

## Abstract

**Background:**

Angiogenesis is essential for tumor growth. Hepatocellular carcinoma (HCC) is characterized by hypervascularity; high levels of angiogenesis are associated with poor prognosis and a highly invasive phenotype in HCC. Up-regulated gene-4 (*URG4*), also known as upregulator of cell proliferation (*URGCP*), is overexpressed in multiple tumor types and has been suggested to act as an oncogene. This study aimed to elucidate the effect of URG4/URGCP on the angiogenic capacity of HCC cells *in vitro*.

**Methods:**

Expression of URG4/URGCP in HCC cell lines and normal liver epithelial cell lines was examined by Western blotting and quantitative real-time PCR. URG4/URGCP was stably overexpressed or transiently knocked down using a shRNA in two HCC cell lines. The human umbilical vein endothelial cell (HUVEC) tubule formation and Transwell migration assays and chicken chorioallantoic membrane (CAM) assay were used to examine the angiogenic capacity of conditioned media from URG4/URGCP-overexpressing and knockdown cells. A luciferase reporter assay was used to examine the transcriptional activity of nuclear factor kappa – light – chain - enhancer of activated B cells (NF-κB). NF-κB was inhibited by overexpressing degradation-resistant mutant inhibitor of κB (IκB)-α. Expression of vascular endothelial growth factor C (*VEGFC*), tumor necrosis factor-α (*TNFα*), interleukin (*IL*)-6, *IL-8* and v-myc avian myelocytomatosis viral oncogene homolog (*MYC)* were examined by quantitative real-time PCR; VEGFC protein expression was analyzed using an ELISA.

**Results:**

URG4/URGCP protein and mRNA expression were significantly upregulated in HCC cell lines. Overexpressing URG4/URGCP enhanced - while silencing *URG4/URGCP* decreased - the capacity of HCC cell conditioned media to induce HUVEC tubule formation and migration and neovascularization in the CAM assay. Furthermore, overexpressing URG4/URGCP increased - whereas knockdown of *URG4/URGCP* decreased - VEGFC expression, NF-κB transcriptional activity, the levels of phosphorylated (but not total) IκB kinase (IKK) and IκB-α, and expression of *TNFα, IL-6, IL-8* and *MYC* in HCC cells. Additionally, inhibition of NF-κB activity in HCC cells abrogated URG4/URGCP-induced NF-κB activation and angiogenic capacity.

**Conclusions:**

This study suggests that URG4/URGCP plays an important pro-angiogenic role in HCC via a mechanism linked to activation of the NF-κB pathway; URG4/URGCP may represent a potential target for anti-angiogenic therapy in HCC.

**Electronic supplementary material:**

The online version of this article (doi:10.1186/s12885-015-1378-7) contains supplementary material, which is available to authorized users.

## Background

Angiogenesis, the formation of new blood vessels, occurs during numerous physiological and pathological processes [1]. Angiogenesis is required to maintain tumor growth and metastasis, and constitutes an important hallmark of tumor progression [2-5]. Tumor angiogenesis is the generation of a network of blood vessels that penetrates into the tumor to supply the nutrients and oxygen required to maintain and enable tumor growth and invasion. Consequently, blocking tumor angiogenesis could prevent the formation of tumor blood vessels and inhibit or slow the growth and spread of tumor cells [6-8]. Angiogenesis is widely regarded to be an effective therapeutic target and promising biomarker for the diagnosis of cancer; therefore, angiogenesis is an important field of research in biological and clinical oncology [9-13]. Tumor angiogenesis is a consequence of an imbalance between pro-angiogenic factors, such as the vascular endothelial growth factor (VEGF) family and IL-8/CXCL8, and inhibitors of angiogenesis, including endostatin, angiostatin and other related molecules [14-16]. VEGF regulates the sprouting and proliferation of endothelial cells and can stimulate tumor angiogenesis [17]. A number of currently-used anti-angiogenesis drugs function by inhibiting pro-angiogenic factors, for example the monoclonal antibody bevacizumab binds to VEGF and prevents it from binding to the VEGF receptors, and sunitinib and sorafenib are small molecules that attach to VEGF-R and inhibit the binding of VEGF [18,19]. However, the precise regulation and mechanisms of tumor angiogenesis are not yet fully explored and the identification of other novel specific, effective inhibitors of angiogenesis is urgently required to treat patients with cancer.

Hepatocellular carcinoma (HCC) accounts for 90% of all primary malignant liver cancers and is the fifth most common cancer and third most common cause of cancer-related mortality worldwide [20,21]. HCC has a much higher morbidity in Asia due to the high incidence of hepatitis B virus (HBV) and hepatitis C virus (HCV) infection, especially in China where 55% of all cases of HCC worldwide occur [21]. HCC is characterized by hypervascularity indicative of angiogenesis, and tumor growth in HCC relies on the formation of new blood vessels [15]. VEGF has been reported to a play critical role in angiogenesis in HCC [22]. Targeting angiogenesis using pharmacologic strategies has recently been validated in several other solid tumor types [23]. Therefore, identification of an anti-angiogenic strategy for HCC may help to improve the treatment outcomes and extend survival for patients with HCC.

Up-regulated gene-4 (*URG4*), also known as upregulator of cell proliferation (*URGCP*), is located on chromosome 7p13 and was identified and initially characterized by Tufan *et al*. URG4/URGCP is upregulated in the presence of hepatitis B virus X antigen (HBxAg) and contributes to the development of HCC as it can promote hepatocellular growth and survival both *in vitro* and *in vivo* [24]. Previous studies demonstrated that URG4/URGCP is upregulated in human HCC and gastric cancer and URG4/URGCP could promote the proliferation and tumorigenicity of HCC and gastric cancer cells [25,26]. Based on these findings, URG4/URGCP has been suggested to function as an oncogene in multiple tumor types [25-28]. However, the effect of URG4/URGCP on tumor angiogenesis in HCC has not yet been elucidated.

In the present study, we demonstrate that URG4/URGCP is upregulated in HCC cell lines. Additionally, ectopic overexpression of URG4/URGCP enhanced the angiogenic capacity of HCC cells *in vitro* and also upregulated VEGF and activated the NF-κB signaling pathway, whereas knockdown of *URG4/URGCP* had the opposite effects. This study demonstrates that URG4/URGCP may promote angiogenesis and the expression of VEGF-C in HCC by activating the NF-κB signaling pathway; therefore, URG4/URGCP may have potential as a therapeutic target in HCC.

## Methods

### Cells and treatments

The normal liver epithelial cell lines Lo2 and THLE3 were purchased from and cultured as recommended by the American Type Culture Collection (Manassas, VA, USA). The HCC cell lines Hep3B, MHCC97H, HepG2, SMMC-7721, QGY-7703, Huh7 and BEL-7402 were purchased from the ATCC and cultured in Dulbecco’s modified Eagle’s medium (DMEM; Invitrogen, Carlsbad, CA, USA) supplemented with 10% fetal bovine serum (FBS) and 100 U penicillin-streptomycin (Invitrogen) in a humidified incubator at 37°C in 5% CO_2_.

### Vectors, retrovirus infection and transfection

The URG4/URGCP expression construct was generated by sub-cloning PCR-amplified full-length human *URG4/URGCP* cDNA into pMSCV-retro-puro (Promega, Madison, WI, USA) using the forward primer 5′-CCAGATCTACCATGG CGTCGCCCGGGCATTC-3′ and reverse primer 5′-GCCGAATTCTCACAGC CGTCTCACCAGCT-3′.

To knockdown *URG4/URGCP*, a siRNA sequence targeting human *URG4/URGCP* (5′-ACCAAAGACTTGCCCTGGAATT-3′; synthesized by Invitrogen) was cloned into pSuper-retro-puro (Promega) to generate pSuper-retro-URG4/URGCP-RNAi (referred to as URG4-Ri) [26]. Retrovirus generation and infection were performed as described previously [29].

The vector pBabe-Puro-IκBα-mut, which expresses degradation-resistant IκBα mutant protein (referred to as IκBα-mut), was purchased from Addgene (plasmid 15291; Cambridge, MA, USA) and used as a NF-κB inhibitor. The HCC cells were transiently transfected with pBabe-Puro-IκBα-mut using Lipofectamine 2000 reagent (Invitrogen) according the manufacturer’s instructions.

### Quantitative real-time RT-PCR

Total cellular RNA was extracted using TRIzol reagent (Invitrogen) and 2 μg of RNA was subjected to cDNA synthesis using random hexamers. Quantitative real-time RT-PCR (qRT-PCR) was performed using an Applied Biosystems 7500 Sequence Detection system with an initial denaturation step at 95°C for 10 min, followed by 28 cycles of denaturation at 95°C for 60 sec, primer annealing at 58°C for 30 sec and primer extension at 72°C for 30 sec, with a final extension step at 72°C for 5 min. Target gene expression was calculated using the threshold cycle (Ct) values and the formula 2^-[(Ct of *Genes*) – (Ct of *GAPDH*)]^ relative to the internal control gene *GAPDH*. PCR primers were designed using Primer Express version 2.0 (Applied Biosystems, Foster City, CA, USA) and were as follows: *VEGFC* forward: 5′-GTGTCCAGTGTAGATGAACTC-3′ and reverse: 5′-ATCTGTAGACGGACACACATG-3′; *TNFα* forward: 5′-CCAGGCAGTCAGATCATCTTCTC-3′ and reverse: 5′-AGCTGGTTATCTCTCAGCTCCAC-3′; *IL-6* forward: 5′-TCTCCACAAGCGCCTTCG-3′ and 5′-CTCAGGGCTGAGATGCCG; *IL-8* forward: 5′-TGCCAAGGAGTGCTAAAG-3′ and reverse: 5′-CTCCACAACCCTCTGCAC-3′; *MYC* forward: 5′-TCAAGAGGCGAACACACAAC-3′ and reverse: 5′-GGCCTTTTCATTGTTTTCCA-3′; *GAPDH* forward: 5′-ATTCCACCCATGGCAAATTC-3′ and reverse: 5′-AGAGGCAGGGATGATGTTCTG-3′.

### Western blotting

Total cellular protein was extracted and the samples were heated at 100°C for 5 min. Samples containing 20 μg protein were separated by SDS-PAGE, electro-blotted onto PVDF membranes (Millipore, Billerica, MA, USA), blocked in non-fat milk, probed with polyclonal rabbit anti-URG4 (Abcam, Cambridge, MA, USA), anti-IKK, anti-phosphorylated-IKK (p-IKK), anti-IκBα or anti-p-IκBα (p-IκBα; all Cell Signaling, Danvers, MA, USA). The membranes were stripped and re-probed using anti-α-Tubulin (Cell Signaling) as a loading control.

### HUVEC tubule formation assay

The HUVEC tubule formation assay was performed as previously reported [23]. Briefly, 200 μl Matrigel was placed into each well of a 24-well plate and polymerized for 30 min at 37°C. HUVECs (approximately 2 × 10^4^) in 200 μl conditioned media (CM) from indicated HCC cells were added to each well and incubated for 24 h at 37°C in 5% CO_2_. Images were captured at 100× using a bright-field microscope, and formation of capillary tubes was quantified by measuring their total length of each image.

### Chicken chorioallantoic membrane assay

The chicken chorioallantoic membrane (CAM) assay was performed using eight-day-old fertilized chicken eggs. A 1 cm diameter window was created in the shell of each egg and the surface of the dermic sheet was removed to expose the CAM. A 0.5 cm diameter filter paper was placed on top of the CAM, and 100 μl CM harvested from the indicated HCC cells placed on the center of the filter paper. The eggs were incubated at 37°C at 80-90% relative humidity for 48 h, then the windows in the shell were closed using sterilize bandages. Following fixation with stationary solution (1:1 *vol/vol* mixture of methanol and acetone) for 15 min, the CAM was excised and imaged using a digital camera. The number of second- and third-order vessels in the test groups was expressed relative to that of CAM treated with CM from the vector control cells.

### HUVEC transwell migration assay

HUVECs (approximately 1 × 10^4^) were plated on the top of polycarbonate Transwell filters (pore size 8.0 μm; Corning Incorporated, Corning, NY, USA ) in CM containing 5% FBS. The lower chamber was filled with 500 μl of media containing 15% FBS. The cells were incubated at 37°C for about 20 h, and the cells that migrated to the lower membrane surface were fixed in 4% paraformaldehyde, stained using hematoxylin for 15 min, and the number of cells in ten randomly-selected 200× fields of view per filter was counted and expressed relative to that of cells treated with CM from vector control cells.

### Luciferase reporter assay of NF-κB transcriptional activity

The pNF-κB-luciferase reporter and control plasmids (Clontech, Mountain View, CA, USA) were used examine NF-κB transcriptional activity. Approximately 1.5 × 10^4^ HCC cells were seeded in triplicate in 24-well plates, allowed to adhere, and co-transfected with 100 ng of the NF-κB luciferase reporter plasmid or control luciferase plasmid and 1 ng of pRL-TK Renilla plasmid (Promega) using Lipofectamine 2000 reagent (Invitrogen). The luciferase and Renilla signals were measured 48 h after transfection using the Dual Luciferase Reporter Assay Kit (Promega) according to the manufacturer’s protocol.

### Enzyme-linked immunosorbent assay

The VEGFC enzyme-linked immunosorbent assay (ELISA) was performed using a commercial kit according to the manufacturer’s instructions (Keygentec Co., Shanghai, China). Briefly, standard solutions, test samples and negative control samples were added to the plate in triplicate, incubated at 36°C for 90 min, washed, incubated with a specific anti-VEGFC antibody (Cell Signaling) at 36°C for 1 h, washed, incubated with secondary antibody from the kit for 1 h, substrate was added, incubated for 1 h and the absorbance values were read at OD_450_ using an ELISA plate reader.

### Statistical analysis

All experimental data are presented as the mean ± SD of three independent biological replicates. Statistical analyses were performed using SPSS 13.0 (IBM, Armonk, NY, USA). Analysis of variance (ANOVA) was used to evaluate the significance of the differences between two groups. *P-*values ≤ 0.05 were considered statistically significant.

## Results

### URG4/URGCP is upregulated in HCC cell lines

**Figure 1:**
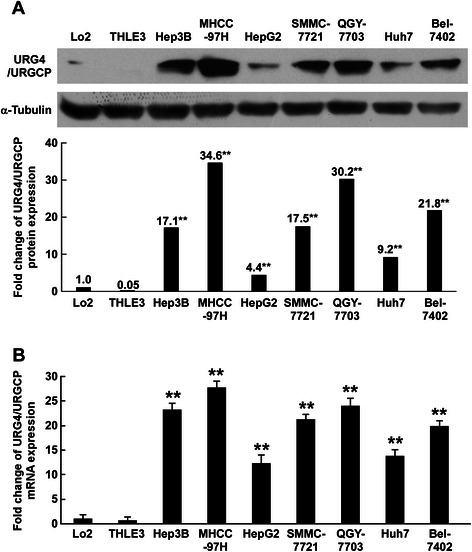
URG4/URGCP is upregulated in HCC cell lines. **A**. Western blotting analysis of URG4/URGCP protein expression in two normal liver cell lines and seven HCC cell lines; α-Tubulin was used as a loading control. Lower panel, quantification of Western blotting data relative to Lo2 cells. **B**. Real-time PCR quantification of *URG4/URGCP* mRNA expression in two normal liver cell lines and seven HCC cell lines. Transcript levels were normalized to *GAPDH* and expressed relative to Lo2 cells. Data is mean ± SD of three independent experiments; ** *P* < 0.01.

Western blotting and qRT-PCR analyses were performed to examine URG4/URGCP protein and mRNA expression in HCC cell lines. URGCP/URG4 protein expression was significantly upregulated in all seven HCC cell lines tested compared to two normal liver epithelial cell lines, Lo2 and THLE3, which expressed low or undetectable levels of URGCP/URG4 (Figure 1A). Consistent with the Western blotting analysis, qRT-PCR demonstrated that *URG4/URGCP* mRNA was markedly upregulated in all seven HCC cell lines compared to the normal liver epithelial cell lines (Figure 1B). These data suggest that URG4/URGCP is upregulated in HCC cells.

### URG4/URGCP promotes the angiogenic capacity and expression of VEGFC in HCC cells

**Figure 2:**
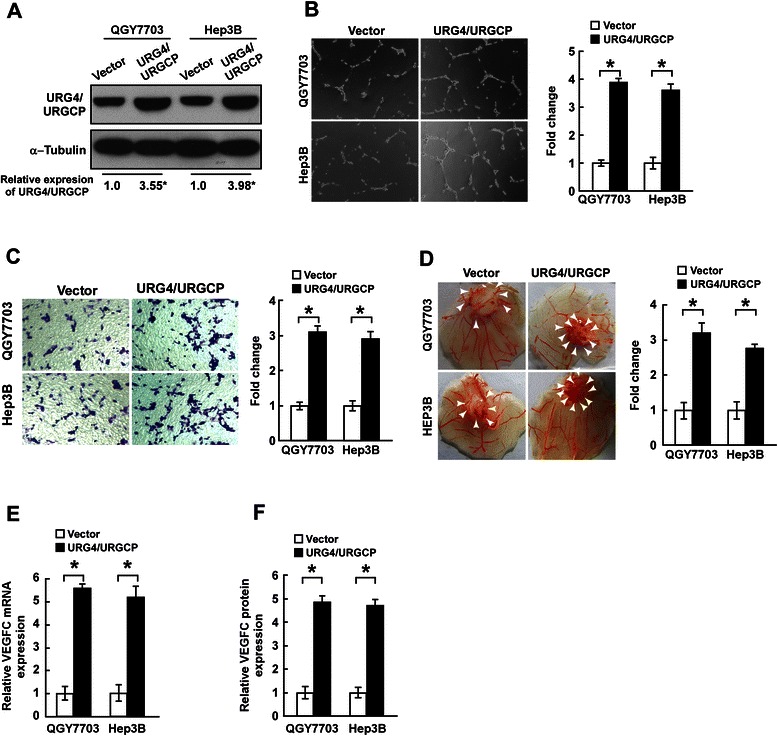
URG4/URGCP enhances the angiogenic capacity of HCC cells *in vitro*. **A**. Western blotting analysis of URG4/URGCP protein expression in QGY7703 - vector, QGY7703-URG4/URGCP, Hep3B-vector and Hep3B-URG4/URGCP cells; α-Tubulin was used as a loading control. The numbers represent the relative expression of each protein compared to the respective control cells. **B**. Representative images (left) and quantification (right) of tube-like structures formed by HUVECs on Matrigel-coated plates when cultured in conditioned medium (CM) derived from the indicated cells. **C**. Representative images (left) and quantification (right) of the number of migrated HUVEC cells after incubation in CM derived from the indicated cells in the Transwell migration assay. **D**. Representative images (left) and quantification (right) of neovessels formed in the CAM assay when stimulated by CM derived from the indicated cells. **E**. Quantitative real-time PCR analysis of *VEGFC* mRNA expression in the indicated cells. Transcript levels were normalized to *GAPDH* and expressed relative to the respective control cells. **F**. ELISA of VEGFC protein expression in the indicated cell supernatants. Data is mean ± SD of three independent experiments; * *P* < 0.05.

The HCC cell lines QGY7703 and Hep3B expressed moderate levels of URG4/URGCP and were used to create stable cell lines overexpressing URG4/URGCP. Overexpression of URG4/URGCP in the stable cell lines was verified by Western blotting (Figure 2A).

Firstly, the effect of URG4/URGCP on the ability of HCC cells to induce angiogenesis was investigated using the HUVEC tubule formation assay. HUVECs were seeded on Matrigel in CM harvested from URG4/URGCP-overexpressing HCC cells. CM derived from URG4/URGCP-transduced cells significantly increased the formation of tubule structures compared to CM from vector control cells (Figure 2B). Moreover, CM from URG4/URGCP-overexpressing HCC cells significantly increased the migration of HUVEC cells in the migration assay (Figure 2C). Furthermore, ectopic overexpression of URG4/URGCP in HCC cells enhanced the ability of CM to induce the formation of second- and third-order vessels in the CAM assay (Figure 2D).

As neovessel formation is closely associated with VEGFC, we examined the expression of VEGFC in URG4/URGCP-overexpressing and vector control HCC cells using qRT-PCR and an ELISA. VEGFC mRNA and protein expression were significantly upregulated in the URG4/URGCP-overexpressing HCC cells (Figure 2, E and F). However, the results were not repeated when these experiments were performed with Lo2 and THLE3 cells stably overexpressing URG4/URGCP (Additional file 1: Figure S1). Taken together, these results suggest that URG4/URGCP enhanced the capacity of HCC cells to induce neovessel formation *in vitro*.

### Silencing URG4/URGCP reduces the angiogenic capacity and expression of VEGFC in HCC cells

**Figure 3:**
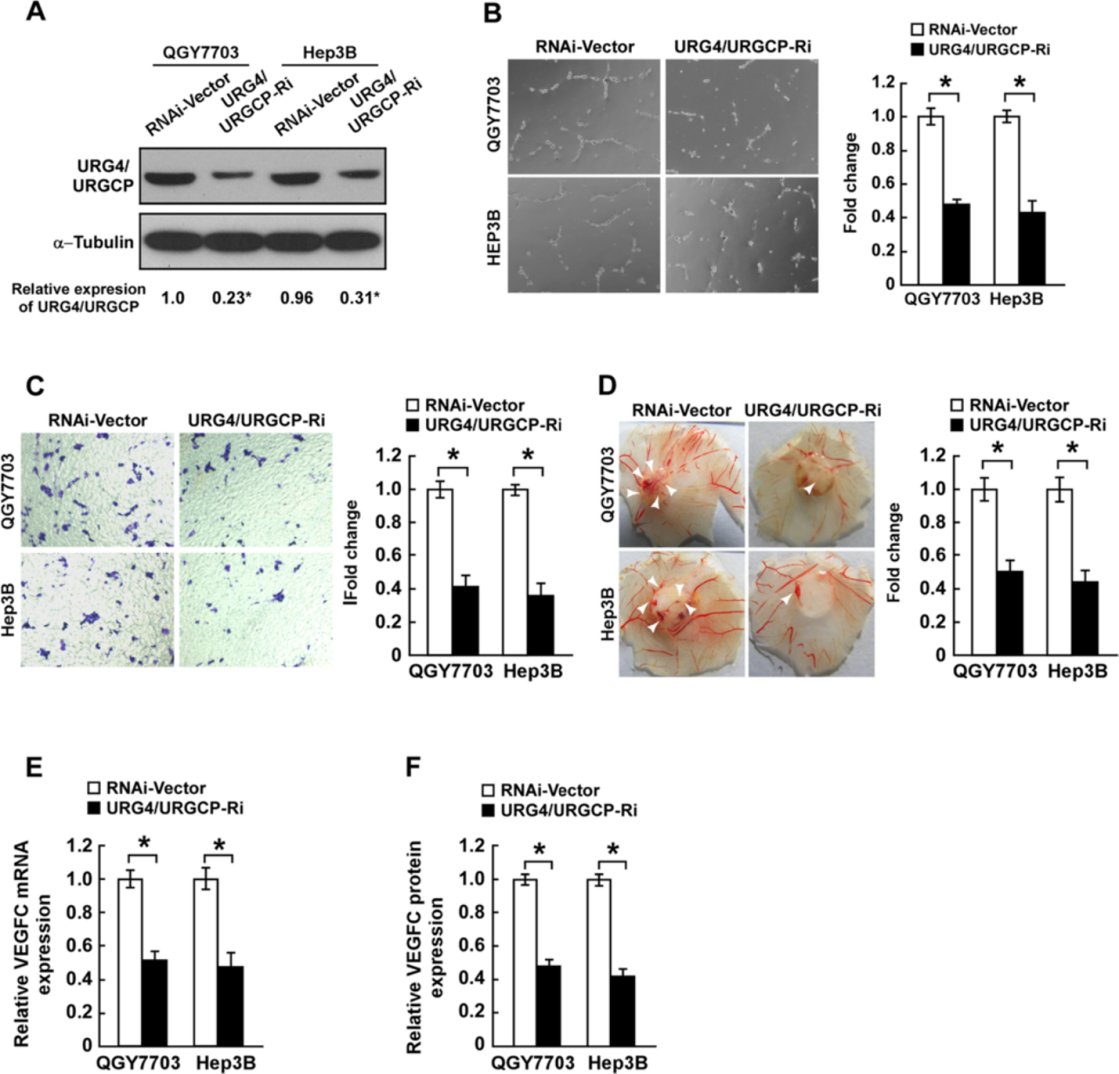
Knockdown of *URG4/URGCP* reduces the angiogenic capacity of HCC cells *in vitro*. **A**. Western blotting analysis of URG4/URGCP protein expression in *URG4/URGCP*-shRNA-transduced QGY7703 and Hep3B cell lines (shown as URG4/URGCP-RNAi) and the corresponding vector control cells; α-Tubulin was used as a loading control. The numbers represent the relative expression of each protein compared to the respective control cells. **B**. Representative images (left) and quantification (right) of tube-like structures formed by HUVECs on Matrigel-coated plates when cultured in conditioned medium (CM) derived from the indicated cells. **C**. Representative images (left) and quantification (right) of the number of migrated HUVEC cells when incubated in CM derived from the indicated cells in the Transwell migration assay. **D**. Representative images (left) and quantification (right) of neovessels formed in the CAM assay when stimulated by CM derived from the indicated cells. **E**. Quantitative real-time PCR analysis of *VEGFC* mRNA expression in the indicated cells. Transcript levels were normalized to *GAPDH* and expressed relative to the respective control cells. **F**. ELISA of VEGFC protein expression in the indicated cell supernatants. Data is mean ± SD of three independent experiments; * *P* < 0.05.

To further confirm the effect of URG4/URGCP on angiogenesis during the progression of HCC, stable QGY7703 and Hep3B cell lines in which *URG4/URGCP* was silenced were established; knockdown of URG4/URGCP in these cells was confirmed by Western blotting (Figure 3A). Compared to CM from vector control cells, CM from URG4/URGCP-silenced cells inhibited tubule formation by HUVECs (Figure 3B), suggesting that knockdown of endogenous URG4/URGCP reduced the ability of HCC cells to promote angiogenesis. Moreover, CM from URG4/URGCP-silenced HCC cells inhibited HUVEC migration (Figure 3C) and decreased the formation of second- and third-order vessels in the CAM assay (Figure 3D). In parallel with these results, knockdown of *URG4/URGCP* significantly reduced VEGFC mRNA and protein expression in both HCC cell lines (Figure 3E and F). These results confirmed that URG4/URGCP enhances the angiogenic capacity of HCC cells.

### URG4/URGCP promotes the angiogenic capacity of HCC cells via activating the NF-κB signaling pathway

**Figure 4:**
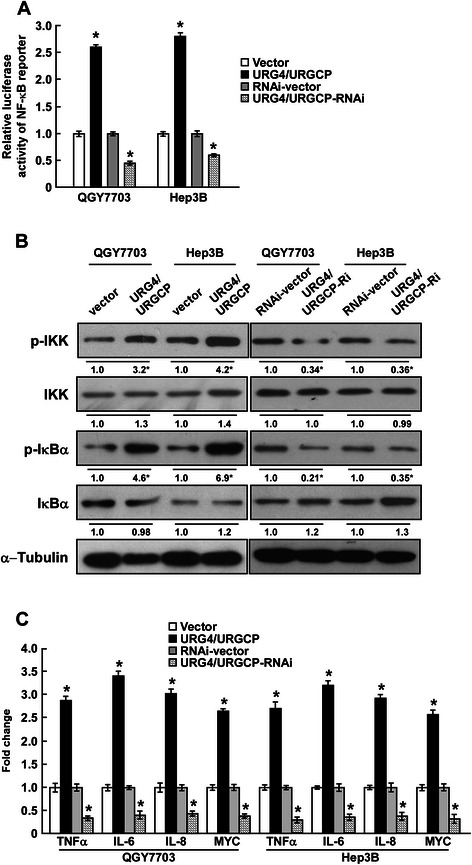
URG4/URGCP promotes NF-κB transcriptional activity. **A**. Luciferase reporter assay of NF-κB transcriptional activity in URG4/URGCP-overexpressing or silenced cells expressed relative to the respective control cells. **B**. Western blotting analysis of the expression of phosphorylated IKK (p-IKK), total IKK, phosphorylated IκBα (p-IκBα) and total IκBα; α-Tubulin was used as a loading control. The numbers represent the relative expression of each protein compared to the respective control cells. **C**. Quantitative real-time PCR analysis of the expression of genes downstream of NF-κB in the indicated cells; transcript levels were normalized to *GAPDH* and expressed relative to the respective vector control cells. Data is mean ± SD of three independent experiments; * *P* < 0.05.

As *VEGFC* has been reported to be a downstream target of the NF-κB pathway [30-33], we explored effect of URG4/URGCP on NF-κB signaling activity. Luciferase reporter assays demonstrated that overexpression of URG4/URGCP enhanced the transcriptional activity of a NF-κB reporter gene, while knockdown of *URG4/URGCP* suppressed NF-κB transcriptional activity (Figure 4A). Western blotting showed that overexpression of URG4/URGCP increased the levels of phosphorylated IKK and phosphorylated IκBα but did not significantly change the total protein level of IKK or IκBα (Figure 4B). In addition, the levels of number of NF-κB target genes, including *TNF-α*, *IL-6*, *IL-8* and *MYC*, were upregulated in URG4/URGCP-overexpressing cells and downregulated in URG4/URGCP-silenced HCC cells (Figure 4C). Taken together, these results indicated that the NF-κB pathway may underlie the pro-angiogenic effect of URG4/URGCP in HCC.

### Inhibition of NF-κB signaling activity inhibits the ability of URG4/URGCP to enhance the angiogenic capacity of HCC cells

**Figure 5:**
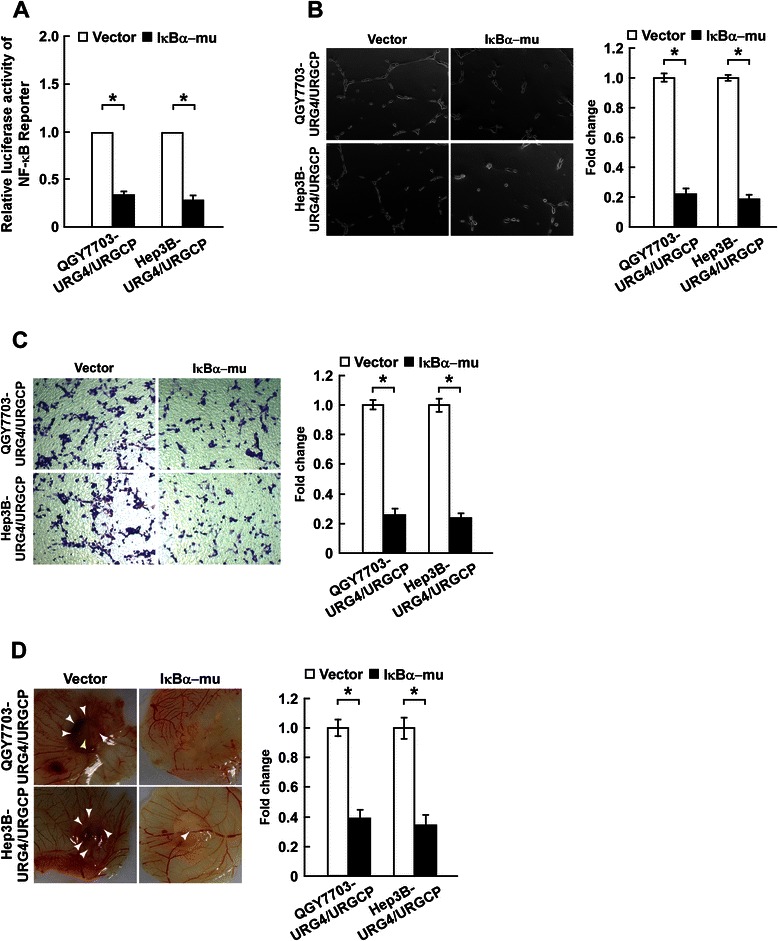
URG4/URGCP enhances the angiogenic capacity of HCC cells via activating the NF-κB pathway. URG4/URGCP-overexpressing HCC cells were transfected with a non-degradable mutant IκBα protein, which acts as a specific NF-κB inhibitor. **A**. Luciferase reporter assay of NF-κB transcriptional activity in the indicated cells. **B**. Representative images (left) and quantification (right) of tube-like structures formed by HUVECs on Matrigel-coated plates in the presence of CM from the indicated cells. **C**. Representative images (left) and quantification (right) of the number of migrated HUVEC cells in the Transwell migration assay after incubation in CM derived from the indicated cells. **D**. Representative images (left) and quantification (right) of neovessels formed in the CAM assay when stimulated by CM derived from the indicated cells. Data is mean ± SD of three independent experiments; *P < 0.05.

We further explored whether URG4/URGCP increased the angiogenic capacity of HCC cells by activating NF-κB signaling. NF-κB signaling was inhibited by transient overexpression of a non-degradable IκBα mutant containing alanine residues in positions 32 and 36 instead of serine residues, which cannot be phosphorylated and degraded [34] and thus remains bound to and inhibits NF-κB. The stimulatory effects of CM derived from URG4/URGCP-overexpressing HCC cells on HUVEC tubule formation and migration were significantly reversed when the IκBα mutant was transiently overexpressed in the HCC cells (Figure 5, A-C; Additional file 2: Figure S2). Similar results were obtained in the CAM assay, as the IκBα mutant reversed the ability of CM collected from URG4/URGCP-overexpressing HCC cells to promote angiogenesis (Figure 5D). Collectively, these data suggest that URG4/URGCP enhances the angiogenic capacity of HCC cells via a mechanism involving functional activation of the NF-κB signaling pathway.

## Discussion

*URG4/URGCP* can promote the growth and survival of HCC cells and was the first gene identified to be upregulated in the presence of HBxAg [24], indicating URG4/URGCP may potentially play a role in the progression of HCC. Besides its ability to promote HCC cell proliferation, the precise role of URG4/URGCP in HCC has not yet been elucidated [24,26]. In this study, we demonstrate for the first time that URG4/URGCP can enhance the angiogenic capacity of HCC cells *in vitro*; therefore, URG4/URGCP may exert a number of functions during the development and progression of HCC and should be considered as a potential novel therapeutic target for HCC. Besides the hepatocarcinogenesis function of URG4/URGCP, it has been reported that URG4/URGCP is also upregulated in gastric cancer tissues and cells and enhances gastric cancer cell proliferation and tumorigenesis [25]. High expression level of URG4 was also found in acute lymphoblastic leukemia (ALL) patients indicating that URG4 might be involved in leukemogenesis [35]. However, future studies are needed to demonstrate the exact role of URG4 in various malagnancies.

Although several studies have indicated that URG4/URGCP may act as an oncogene in various tumor types [26,36-38], the exact function and molecular mechanism of actions of URG4/URGCP have not been precisely characterized. In the present study, we found that overexpression of URG4/URGCP increased the formation of tubule structures in HUVEC cells and significantly increased the migration of HUVEC cells in the migration assay, and enhanced the ability to induce the formation of second- and third-order vessels of CAM. All of the results indicate the promotive effect of URG4/URGCP in HCC angiogenic progression. In combination with the ability of URG4/URGCP to promote the angiogenic capacity of HCC cells, VEGFC was markedly upregulated in URG4/URGCP-overexpressing cells, indicating that an association exists between URG4/URGCP and VEGFC. *VEGFC* is one of the target genes downstream of the NF-κB pathway [30-33]. Luciferase reporter assays showed overexpression of URG4/URGCP significantly enhanced the transcriptional activity of NF-κB, suggesting NF-κB plays an essential role in the URG4/URGCP-induced angiogenic capacity of HCC cells. NF-κB has been widely studied as a transcription factor that regulates inflammatory and immune responses, as well as range of other physiological and pathological processes including the development and progression of cancer [39,40]. Aberrant activation of NF-κB is observed in a variety of tumor types. NF-κB mediates a range of biological processes in cancer cells by transcriptionally activating numerous target genes [41,42]. Activation of NF-κB signaling is negatively regulated by the IκBs, which bind and sequester NF-κB in the cytoplasm in an inactive state. IκBs are phosphorylated by IKKs, which leads to ubiquitin-mediated degradation of the IκBs and consequently enables the release and translocation of NF-κB to the nucleus [43-45]. Consistent with these well-studied processes, the present study demonstrated that overexpression of URG4/URGCP upregulated the level of p-IKK and p-IκBα and ultimately enhanced the activation of NF-κB. Additionally, when the cells overexpressing URG4/URGCP were transfected with the IκBα mutant, the capacity of CM from URG4/URGCP-overexpressing cells to enhance the angiogenic capacity of HCC cells was attenuated. These findings indicate that URG4/URGCP promotes the angiogenic capacity of HCC cells - at least in part - by activating the NF-κB/VEGFC signaling pathway.

Additionally, overexpression of URG4/URGCP upregulated a number of genes downstream of the NF-κB signaling pathway: *TNF, IL-6, IL-8* and *MYC*. TNF-α is well-recognized to promote angiogenesis and drive remodeling of blood vessels *in vivo* [46-48]; interleukin-6 increases the expression of VEGF and can promote angiogenesis [49-51]; IL-8 has been shown to play an important role in tumor angiogenesis [52]; and Myc plays an essential role in vasculogenesis and angiogenesis during the development and progression of various types of cancer [53-55]. It would be interesting to explore whether *TNF, IL-6, IL-8* or *MYC* play a role in angiogenesis and disease progression in HCC, and explore the correlation between the expression of these genes and *VEGFC*. The regulatory mechanism by which upregulation of URG4/URGCP modulates the NF-κB/VEGFC pathway and enhances the angiogenic capacity of HCC cells remains to be elucidated and should be investigated further.

## Conclusion

In conclusion, this study demonstrates that URG4/URGCP is upregulated in HCC cell lines and enhances the angiogenic capacity of HCC cells via activation of the NF-κB signaling pathway. These results may provide new insight into the mechanisms that regulate angiogenesis in HCC; targeting URG4/URGCP may represent a promising therapeutic strategy for HCC.

## Additional files

Additional file 1: Figure S1. Effect of URG4/URGCP on the angiogenic capacity of normal hepatic cell lines. A. Western blotting analysis of URG4/URGCP protein expression in Lo2 and THLE3 cells transduced with either pMSCV-URG4/URGCP or the control vector pMSCV; α-Tubulin was used as a loading control. B. Representative images (left) and quantification (right) of tube-like structures formed by HUVECs cultured on Matrigel-coated plates in the presence of CM from the indicated cells. C. Representative images (left) and quantification (right) of the number of migrated HUVEC cells in the Transwell migration assay after incubation in CM derived from the indicated cells. D. Representative images (left) and quantification (right) of neovessels formed in the CAM assay when stimulated by CM derived from the indicated cells. E. Quantitative real-time PCR analysis of *VEGFC* mRNA expression in the indicated cells; transcript levels were normalized to *GAPDH* and expressed relative to the respective vector control cells. F. ELISA of VEGFC protein expression in the indicated cell supernatants. Data is mean ± SD of three independent experiments; * *P* < 0.05.
Additional file 2: Figure S2. Western blotting analysis of phosphorylated IκBα expression in the indicated cells; α-Tubulin was used as a loading control.

## Abbreviations

HCC: hepatocellular carcinoma
URG4: up-regulated gene-4
URGCP: upregulator of cell proliferation
NF-κB: nuclear factor kappa-light-chain-enhancer of activated B cells
VEGF: vascular endothelial growth factor
PDGF: platelet-derived growth factor
FGFs: fibroblast growth factors
IκB: inhibitor of kappa B
IKK: IκB kinase
HUVEC: human umbilical vein endothelial cells
CAM: chicken chorioallantoic membrane
IL: interleukin
TNF-α: tumor necrosis factor alpha
HBV: hepatitis B virus
HCV: hepatitis C virus
HBxAg: hepatitis B virus X antigen
DMEM: Dulbecco’s modified Eagle’s medium
ATCC: American Type Culture Collection
FBS: fetal bovine serum
qRT-PCR: quantitive real-time RT-PCR
SD: standard deviation
