# Supplementary figures and images for: URG4/URGCP enhances the angiogenic capacity of human hepatocellular carcinoma cells in vitro via activation of the NF-κB signaling pathway
Source: BMC Cancer. 2015 May 7;15:368. doi: 10.1186/s12885-015-1378-7 (PMC4437676; doi:10.1186/s12885-015-1378-7)

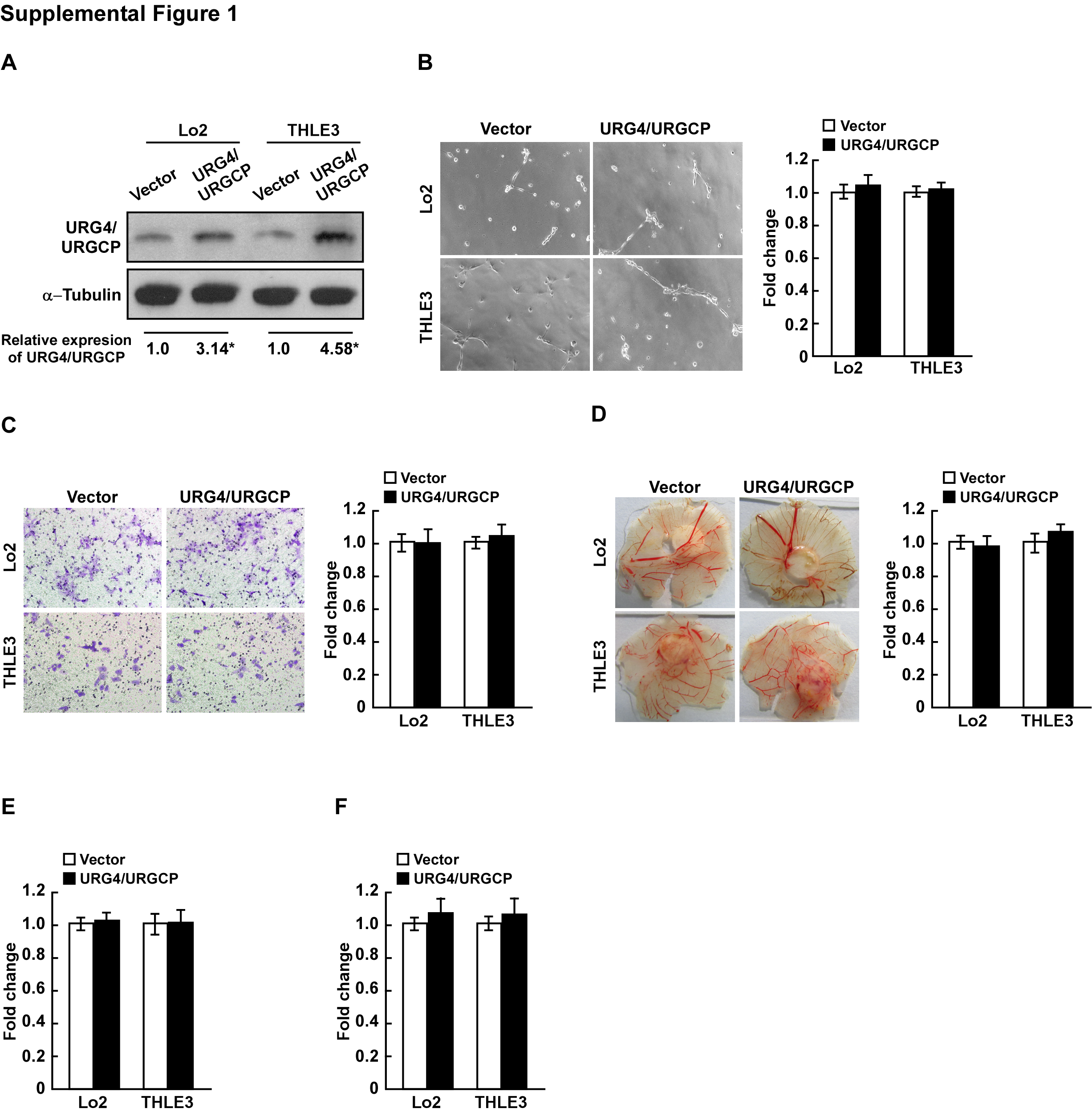

Supplement: Additional file 1: Figure S1. — Effect of URG4/URGCP on the angiogenic capacity of normal hepatic cell lines. A. Western blotting analysis of URG4/URGCP protein expression in Lo2 and THLE3 cells transduced with either pMSCV-URG4/URGCP or the control vector pMSCV; α-Tubulin was used as a loading control. B. Representative images (left) and quantification (right) of tube-like structures formed by HUVECs cultured on Matrigel-coated plates in the presence of CM from the indicated cells. C. Representative images (left) and quantification (right) of the number of migrated HUVEC cells in the Transwell migration assay after incubation in CM derived from the indicated cells. D. Representative images (left) and quantification (right) of neovessels formed in the CAM assay when stimulated by CM derived from the indicated cells. E. Quantitative real-time PCR analysis of VEGFC mRNA expression in the indicated cells; transcript levels were normalized to GAPDH and expressed relative to the respective vector control cells. F. ELISA of VEGFC protein expression in the indicated cell supernatants. Data is mean ± SD of three independent experiments; * P < 0.05. [file 12885_2015_1378_MOESM1_ESM.tiff]

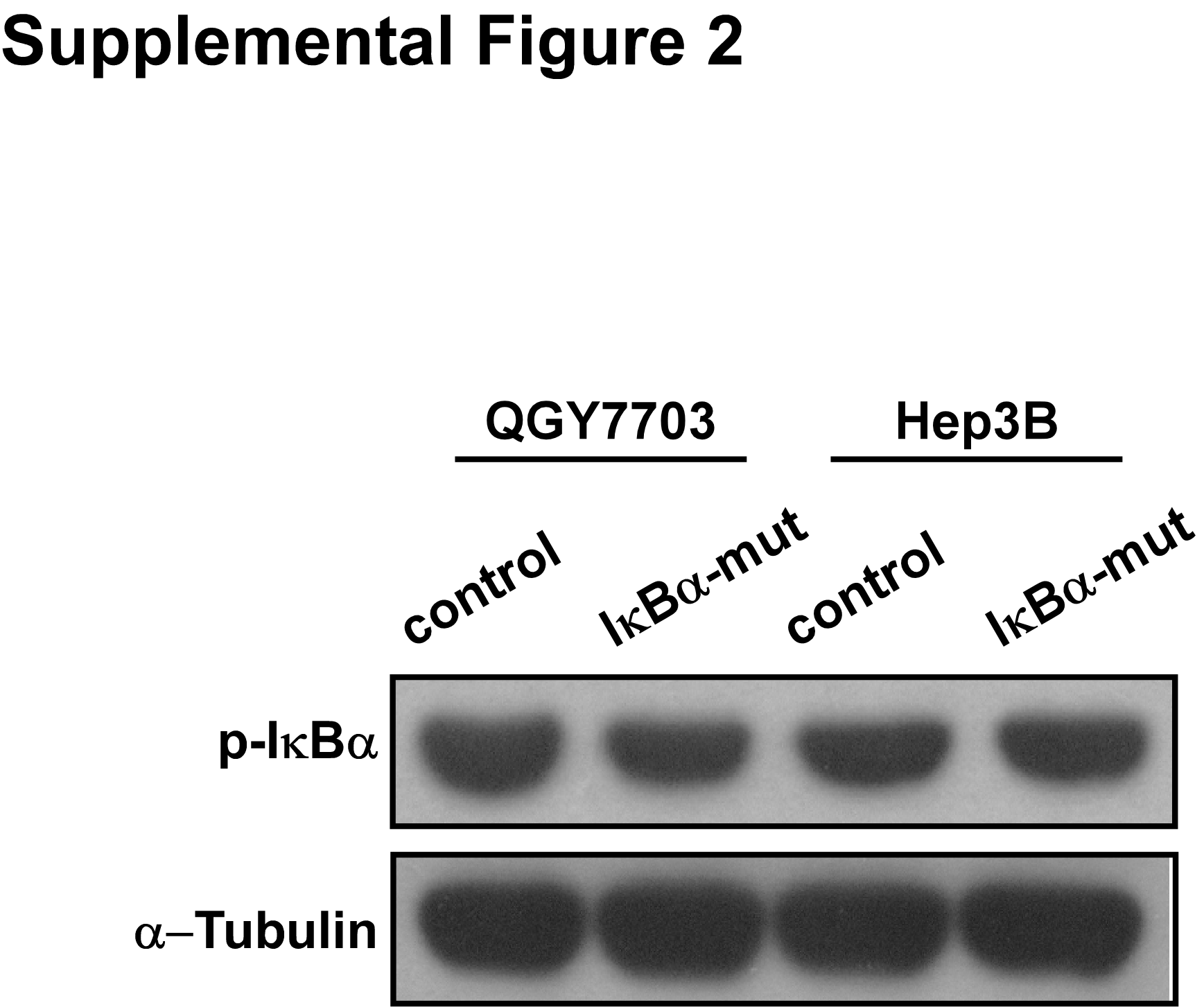

Supplement: Additional file 2: Figure S2. — Western blotting analysis of phosphorylated IκBα expression in the indicated cells; α-Tubulin was used as a loading control. [file 12885_2015_1378_MOESM2_ESM.tiff]
